# Correlation between KRAS mutation subtypes and prognosis in Chinese advanced non‐squamous non‐small cell lung cancer patients

**DOI:** 10.1002/cam4.5995

**Published:** 2023-05-04

**Authors:** Feiwen Liu, Fang Wang, Jianbo He, Shaozhang Zhou, Min Luo

**Affiliations:** ^1^ The Third Affiliated Hospital of Guangxi Medical University Nanning City Guangxi Zhuang Autonomous Region China; ^2^ Guangxi Qianhai Life Hospital Nanning City Guangxi Zhuang Autonomous Region China; ^3^ Department of Respiratory Oncology Guangxi Medical University Cancer Hospital Guangxi Zhuang Autonomous Region Nanning City China

**Keywords:** combined therapy, KRAS mutation, non‐squamous NSCLC, overall survival, progression‐free survival

## Abstract

**Purpose:**

The relationship between mutant KRAS and the risk of disease progression and death in advanced non‐squamous non‐small cell lung cancer (NSCLC) is still controversial among current studies, and the effects of distinct KRAS mutations on prognosis may be different. This study aimed to further investigate the association between them.

**Patients and Methods:**

Of the 184 patients eventually included in the study, 108 had KRAS wild type (WT) and 76 had KRAS mutant type (MT). Kaplan–Meier curves were plotted to describe the survival for patients among groups, while log‐rank tests were conducted to evaluate the survival differences. The univariate and multivariate Cox regression were performed to identify predictors, and subgroup analysis was used to verify the interaction effect.

**Results:**

Similar efficacy of first‐line therapy was observed for KRAS MT and WT patients (*p* = 0.830). The association between KRAS mutation and progression‐free survival (PFS) was not significant in univariate analysis (hazard ratio [HR] = 0.94; 95% CI, 0.66–1.35), and no KRAS mutation subtype significantly affected PFS. However, KRAS mutation and KRAS non‐G12C were associated with increased risk of death compared to KRAS WT in univariate and multivariate analysis. Univariate and multivariate analysis also confirmed that chemotherapy combined with antiangiogenesis or immunotherapy in the KRAS mutation group was associated with decreased risk of disease progression. However, the overall survival (OS) among KRAS mutant patients received different first‐line treatments did not significantly differ.

**Conclusion:**

KRAS mutations and their subtypes are not independent negative predictors of PFS, while KRAS mutation and KRAS non‐G12C were independent prognostic factors for OS. Chemotherapy combined with antiangiogenesis or immunotherapy conferred decreased risk of disease progression to KRAS mutation patients compared to single chemotherapy.

## Graphical abstract

There was no significant difference in the efficacy of first‐line therapy between KRAS mutation and wild‐type patients. The association between KRAS mutation and progression‐free survival was not significant, and no subtype of KRAS mutation significantly affected the progression‐free survival. KRAS mutation, KRAS G12D, KRAS G12V, and other rare KRAS mutation subtypes were significantly associated with poorer overall survival than KRAS wild‐type. Chemotherapy combined with antiangiogenesis or immunotherapy in the KRAS mutation group was significantly associated with improved progression‐free survival. Patients with KRAS mutation tend to benefit more for progression‐free survival from chemotherapy combined with antivascular therapy or immunotherapy compared to those with KRAS wild‐type.

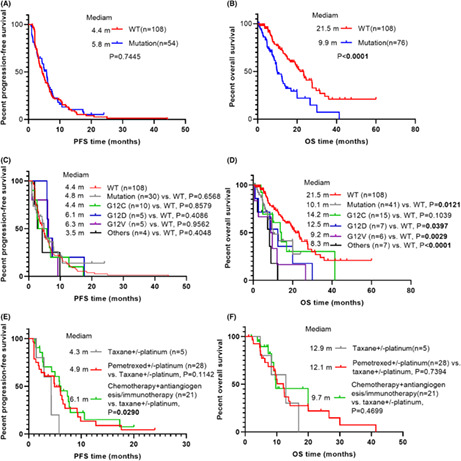

## 1 INTRODUCTION

KRAS missense mutations were the second most frequent oncogenic drivers after epidermal growth factor receptor (EGFR) mutations for non‐small cell lung cancer (NSCLC) in Chinese population.^1^ Approximately 1/4 to 1/3 of NSCLC patients in the Western population had KRAS mutation,^2^, ^3^ but the overall incidence (9.8–12.3%) of KRAS mutations in Chinese population was lower.^1^, ^4^, ^5^ Among the amino acid substitutions, KRAS G12C (29.6%–40%) was the most common in Chinese patients, followed by G12D, G12V and G12A.^5^ In the Chinese lung squamous carcinoma patients, the prevalence of KRAS mutations was 0%–4.5%.^6^, ^7^, ^8^ The mutated KRAS were so rare in squamous cell carcinoma that Rekthman et al. have suggested that KRAS mutations originated from adenocarcinoma cells misclassified as squamous cells or adenocarcinoma components in adenosquamous carcinoma.^9^

Since the discovery of KRAS oncogene in 1982,^10^ the research and development of targeted drugs directly targeting KRAS mutations often ended in failure over the last few decades mainly due to the lack of hydrophobic pockets on the smooth surface of mutant proteins for small‐molecule drugs to bind.^11^ Other targeted agents included inhibitors that inhibited tyrosine kinase receptors, pan‐KRAS proteins, farnesyl transferase, and key proteins lie downstream pathways (e.g., RAF–MEK–ERK and PI3K‐AKT–mTOR signaling pathways) of KRAS protein.^12^, ^13^, ^14^, ^15^, ^16^ Further clinical application of these targeted agents was limited by their low antitumor activity or high incidence of serious adverse events. Biological heterogeneity of KRAS‐mutated tumors, co‐existence of other tumor suppressor genes inactivation, incomplete inhibition of KRAS molecular pathway, and subsequent activation of some signaling feedback loops may be the causes why targeted therapy for KRAS mutation NSCLC is not druggable at present.^17^

Recently, two targeted agents AMG510 and MRTX849 that inhibit specific KRAS G12C mutant protein have presented encouraging outcomes in respective phase II clinical trials, breaking the perceptions that KRAS was considered a undruggable target. Based on the results of phase II clinical trials CodeBreak‐100 and KRYSTAL‐1, AMG510 and MRTX849 are pending regulatory approval for advanced NSCLC patients harboring KRAS G12C mutation and have received at least one regimen of systemic chemotherapy.^18^, ^19^ However, there are still many other KRAS mutant subtypes that do not have effective targeting agents, and current drug availability limits the clinical use of KRAS G12C inhibitors. Importantly, there are currently no randomized controlled trials of AMG510 or MRTX849 for accessing actual efficacy and tolerability at the setting of first‐line treatment in KRAS‐mutated NSCLC.

Therefore, the present treatment methods for advanced NSCLC patients with KRAS mutation are mainly combined modality therapy based on systemic chemotherapy. However, the associations between KRAS mutations and the efficacy of first‐line cytotoxic drugs and overall survival are still controversial, and an in vitro test has shown that distinct KRAS mutations have different sensitivities to different cytotoxic drugs.^20^ Accordingly, the present study hypothesized that different KRAS mutations would lead to different clinical outcomes, and further explored the influence of different treatment regimens on the prognosis of non‐squamous NSCLC patients with KRAS mutation.

## 2 MATERIALS AND METHODS

### 2.1 Study design and population

The present study was conducted as a retrospective cohort study. We consecutively enrolled advanced non‐squamous NSCLC patients who were first admitted to Guangxi Medical University Affiliated Tumor Hospital from March 2016 to September 2020 for further screening. All non‐squamous NSCLC patients participated in this study received standard chemotherapy or chemotherapy‐based combination therapy as per relevant guidelines and clinical norms. Exclusion criteria were as follow: patients at early stages who could receive radical surgery; histological types of squamous cell carcinoma or small‐cell lung cancer; incomplete medical records; no treatment given after diagnosis; KRAS gene mutational status was unknown; detected druggable oncogenic driver mutation; second primary tumor. Figure S1 illustrates in detail the screening and selection process of eligible patients. Ultimately, a total of 184 patients were enrolled in this study in accordance to the inclusion and exclusion criteria. This study was approved by the Ethics Committees of Guangxi Medical University Affiliated Tumor Hospital and followed the Helsinki Guidelines. We only collect the clinical information of the patients, which would not interfere with the treatment decision and harm patients' right or health. In addition, all the patients' data were stored in a coded and anonymous manner. Therefore, no informed consent was necessary.

### 2.2 KRAS mutational assessment

Formalin‐fixed and paraffin‐embedded (FFPE) tissue blocks were used for detecting KRAS mutations by the methods of real‐time polymerase chain reaction (rt‐PCR) or next‐generation sequencing (NGS). Before conducting rt‐PCR analysis, DNA extraction was completed via commercial AmoyDx FFPE DNA Kit (ADx‐FF01, Amoy Diagnostic, Xiamen, China). A NanoDrop 2000 Spectrophotometer (Thermo Scientific, Wilmington, USA) was employed for measuring the purified DNA quantity and quality. Qualitative diagnosis of KRAS mutation was confirmed by the AmoyDx KRAS/NRAS/PIK3CA/BRAF (KNPB) Mutations Detection Kit (ADx‐KNPB01, Amoy Diagnostic, Xiamen, China). The rt‐PCR conditions were as manufacturer's recommended protocols: an initial denaturation at 95°C for 5 min was followed by 15 cycles at 95°C for 25 s, 64°C for 20 s, 72°C for 20 s; and 31 cycles at 93°C for 25 s, 60°C for 35 s, 72°C for 20 s. As for NGS tests, the KRAS mutations data were provided by Nanjing Geneseeq and Beijing Geneplus Technology Inc. in China.

### 2.3 Data collection

KRAS mutations status were detected before first‐line treatment. Specific mutant subtypes cannot be identified when rt‐PCR method was used for detection because of the limitation of KRAS diagnostic kit. OS was calculated as a period from the time of pathological diagnosis until death or last follow‐up, and PFS was recorded as the time from pathological diagnosis to disease progression or death from any cause or follow‐up deadline. The longest follow‐up time was 5 years and cutoff date for follow‐up was March 31st, 2021. We collected clinicopathological characteristics at baseline, including sex, age, body mass index (BMI), Eastern Cooperative Oncology Group performance status (ECOG PS), smoking history, pathological diagnosis, clinical stage, and distant metastases information. The first‐line treatment regimens were obtained for further analyses as well. ECOG PS was applied to assess patients' physical conditions prior to treatment. Non‐smokers were defined as subjects who smoked fewer than 100 cigarettes or never smoked before initial diagnosis. Tumor histological classification was diagnosed by physicians from the Department of Pathology according to the 4th edition WHO classification of lung tumor. Clinical stage was divided through the 8th edition of American Joint Committee on Cancer (AJCC) staging system, while the Response Evaluation Criteria in Solid Tumor (RECIST) version 1.1 was used for evaluating first‐line treatment efficacy.

### 2.4 Statistical analysis

Patients were divided into two groups, KRAS mutation and KRAS WT, in accordance with KRAS mutation status detected before the initial treatment. The chi‐square test or Fisher's exact probability test was performed to identify the associations between KRAS mutation and potential predictors. Kaplan–Meier method was used to present the survival curves, meanwhile log‐rank test was used to compare the survival differences among the groups. Univariate and multivariate Cox proportional hazards models were computed to evaluate the effects of the candidate predictors on clinical outcomes. Sensitivity analyses were illustrated to test the stability of conclusions by forest plots, and interaction was tested to identify heterogeneity among subgroups. Statistical software R packages, version 3.4.3 (http://www.R‐project.org) as well as EmpowerStats (http://www.empowerstats.com) were used for conducting all the analyses. A two‐sided *p* value less than 0.05 were considered as statistically significant in statistical analyses.

## 3 RESULTS

### 3.1 Selected patients and baseline characteristics

**FIGURE 1:**
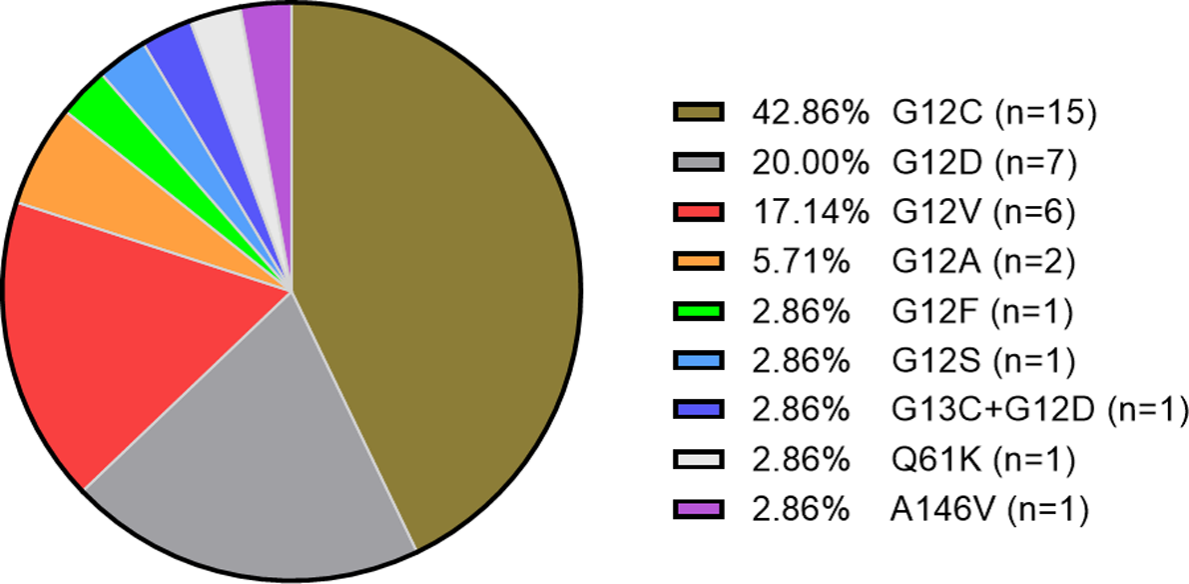
Pie chart of distribution of KRAS mutation in non‐squamous NSCLC patients.

We ultimately screened 184 patients with non‐squamous NSCLC for our final data analysis, including 76 participants with KRAS mutations and 108 participants with KRAS WT. The age range of the study population was 57.2 ± 10.4 years. With a median follow‐up of 20.0 months (95% CI, 17.2–22.6 months), the median durations of PFS and OS for all the patients were 4.8 months (95% CI, 4.2–5.9 months) and 16.6 months (95% CI, 12.9–21.5 months), respectively. Among the 76 cases of KRAS mutation, 41 cases failed to obtain specific amino acid substitution information due to the use of ARMS‐PCR test or data missing. Among the remaining 35 cases, the most frequent KRAS mutation subtypes were G12C, G12D and G12V in exon 2 which accounted 80% of all the mutations, and the remaining mutant subtypes were classified as rare KRAS mutations due to low mutation frequency (Figure 1). Of note, one double mutation of G12D + G13C in exon 2, one Q61K mutation in exon 3, and one A146V mutation in exon 4 were detected. The baseline description of study population was displayed in Table 1. KRAS mutation was closely associated with male, older age, smoking, and adrenal metastases. A lower proportion of IV stage disease and contralateral lung metastases were significantly associated with KRAS mutations as well. As for the first‐line treatment regimens, patients with KRAS mutations are more likely to receive chemotherapy in combination with antiangiogenesis or immunotherapy regimens in clinical management, whereas patients in KRAS wild‐type were more inclined to receive regimens containing pemetrexed or taxane. Other patient characteristics at baseline between KRAS mutation and wild‐type groups were well‐matched.

**TABLE 1:** Patient characteristics at baseline.

	KRAS WT (*n* = 108)	KRAS mutation (*n* = 76)	*p*‐value
Sex			**0.035**
Female	28 (25.93%)	10 (13.16%)	
Male	80 (74.07%)	66 (86.84%)	
Age(years)			**<0.001**
≤60	78 (72.22%)	32 (42.11%)	
>60	30 (27.78%)	44 (57.89%)	
Smoking status			**<0.001**
No	50 (46.30%)	14 (18.42%)	
Yes	58 (53.70%)	62 (81.58%)	
BMI			0.146
≥18.5	90 (83.33%)	69 (90.79%)	
<18.5	18 (16.67%)	7 (9.21%)	
ECOG‐PS			0.597
0–1	97 (89.81%)	70 (92.11%)	
2–3	11 (10.19%)	6 (7.89%)	
Histological type			0.773
Adenocarcinomas	102 (94.44%)	71 (93.42%)	
Others	6 (5.56%)	5 (6.58%)	
Stage			**0.024**
III	10 (9.26%)	16 (21.05%)	
IV	98 (90.74%)	60 (78.95%)	
Brain metastases			0.961
No	87 (80.56%)	61 (80.26%)	
Yes	21 (19.44%)	15 (19.74%)	
Contralateral lung metastases			**0.019**
No	63 (58.33%)	57 (75.00%)	
Yes	45 (41.67%)	19 (25.00%)	
Pleural metastases			0.660
No	72 (66.67%)	53 (69.74%)	
Yes	36 (33.33%)	23 (30.26%)	
Pericardial metastases			0.073
No	98 (90.74%)	74 (97.37%)	
Yes	10 (9.26%)	2 (2.63%)	
Liver metastases			0.814
No	91 (84.26%)	65 (85.53%)	
Yes	17 (15.74%)	11 (14.47%)	
Adrenal metastases			0.062
No	95 (87.96%)	59 (77.63%)	
Yes	13 (12.04%)	17 (22.37%)	
Bone metastases			0.474
No	64 (59.26%)	49 (64.47%)	
Yes	44 (40.74%)	27 (35.53%)	
Number of metastatic sites			0.536
<2	49 (45.37%)	38 (50.00%)	
≥2	59 (54.63%)	38 (50.00%)	
First‐line treatment			**<0.001**
Taxanes ± platinum	16 (14.81%)	5 (6.58%)	
Pemetrexed ± platinum	74 (68.52%)	28 (36.84%)	
Chemotherapy + antiangiogenesis/immunotherapy	13 (12.04%)	21 (27.63%)	
Unknown	5 (4.63%)	22 (28.95%)	

*Note*: *p* values were calculated through the chi‐square test or Fisher's exact test. *p* values < 0.05 were highlighted in bold.

Abbreviations: BMI, body mass index; ECOG PS, Eastern Cooperative Oncology Group Performance Status.

### 3.2 Effect of KRAS mutation on efficacy and PFS

**FIGURE 2:**
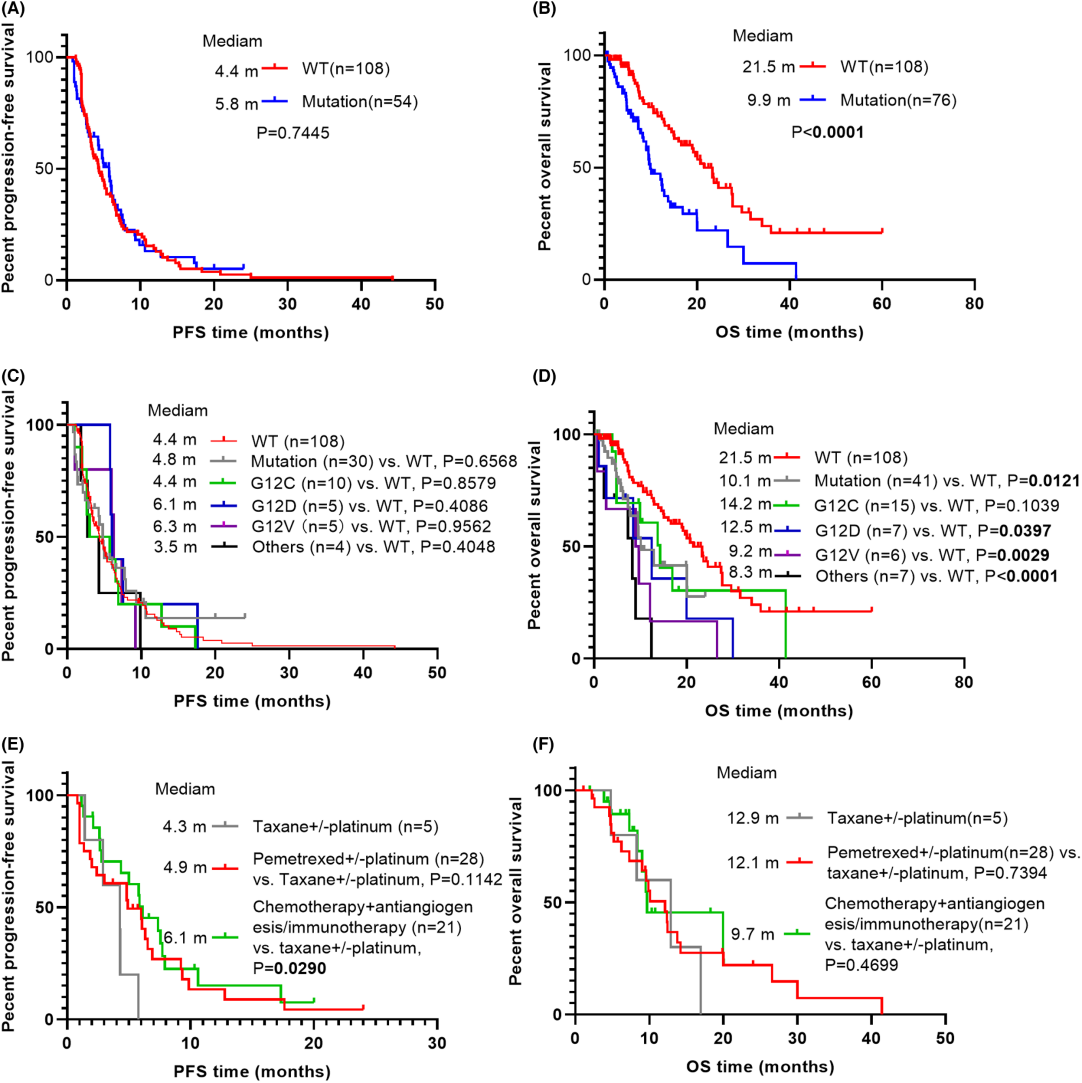
Kaplan–Meier (KM) curves according to different prognostic factors. (A) progression‐free survival curves of all patients stratified by KRAS status; (B) overall survival curves of all patients stratified by KRAS status; (C) progression‐free survival curves of all patients stratified by KRAS mutation subtypes; (D) overall survival curves of all patients stratified by KRAS mutation subtypes; (E) progression‐free survival curves of KRAS‐mutated patients stratified by first‐line treatment; (F) overall survival curves of KRAS‐mutated patients stratified by first‐line treatment.

Response rates for first‐line treatment of study participants were listed in Table 2. First‐line treatment response did not differ significantly in KRAS wild type patients compared to KRAS mutant patients (*p* = 0.830). A total ORR of 25.93% and 30.19%, and a total DCR of 70.37% and 73.58% were observed in KRAS wild‐type and mutant patients, respectively. Furthermore, response rates of KRAS WT and mutant patients were not significantly different no matter what treatment regimen was administered at the first‐line setting. On the other hand, the univariate and multivariate analysis for identifying the predictors for PFS was shown in Table 3. Univariate analysis revealed neither KRAS mutation nor distinct KRAS mutation subtypes significantly affected PFS. No significant difference was observed for median PFS between KRAS WT patients and KRAS mutant patients (4.4 months vs. 5.8 months, *p* = 0.7445) (Figure 2A). Furthermore, patients with specific KRAS mutant subtypes did not have a significantly shorter median PFS than patients with KRAS WT as well (Figure 2C). In addition, the multivariate analysis demonstrated that male, metastatic sites ≥2 and the treatment regimen chemotherapy plus antiangiogenesis or immunotherapy were independent prognostic factors for PFS in both non‐squamous NSCLC patients and non‐squamous NSCLC patients with KRAS mutation. In other words, Chemotherapy in combination with antivascular therapy or immunotherapy did benefit non‐squamous NSCLC patients significantly in PFS regardless of KRAS mutation status. Correspondingly, chemotherapy combined with antivascular therapy or immunotherapy resulted in a longer median PFS compared with taxane‐based chemotherapy, and tended to be correlated with better median PFS compared with pemetrexed‐based chemotherapy in KRAS mutation group (Figure 2E). Interestingly, the effect size of the risk of disease progression in KRAS‐mutated populations (HR = 0.24, 95% CI, 0.08–0.75, *p* = 0.0145) using antivascular or immunotherapy combined with chemotherapy was lower than that in the general population (HR = 0.49, 95% CI, 0.27–0.91, *p* = 0.0233), suggesting that KRAS mutant population may benefit more from combination therapy.

**TABLE 2:** Efficacy analysis of first‐line treatment for non‐squamous NSCLC patients.

	WT (*n* = 108)	Mutation (*n* = 53)	*p*‐values
Total response			0.830
PR	28 (25.93%)	16 (30.19%)	
SD	48 (44.44%)	23 (43.39%)	
PD	32 (29.63%)	14 (26.42%)	
Pemetrexed ± platinum			0.713
PR	18 (24.32%)	7 (25.00%)	
SD	32 (43.25%)	11 (39.29%)	
PD	24 (32.43%)	10 (35.71%)	
Taxanes ± platinum			0.351
PR	2 (12.50%)	1 (20.00%)	
SD	8 (50.00%)	4 (80.00%)	
PD	6 (37.50%)	0 (0.00%)	
Chemotherapy + antiangiogenesis/immunotherapy			0.790
PR	6 (46.15%)	8 (40.00%)	
SD	6 (46.15%)	8 (40.00%)	
PD	1 (7.70%)	4 (20.00%)	

*Note*: *p* values were calculated through the chi‐square test or Fisher's exact test. *p* values < 0.05 were highlighted in bold.

Abbreviations: PD, progressive disease; PR, partial response; SD, stable disease.

**TABLE 3:** Univariate and multivariate analyses of potential predictive factors of PFS.

	^a^Univariate analyses HR (95% CI)	*p*‐value	^a^Multivariate analyses HR (95% CI)	*p*‐value	^b^Univariate analyses HR (95% CI)	*p*‐value	^b^Multivariate analyses HR (95% CI)	*p*‐value
Male	1.59 (1.04, 2.44)	**0.0331**	1.72 (1.09, 2.70)	**0.0187**	2.69 (1.03, 7.03)	**0.0433**	3.93 (1.38, 11.21)	**0.0106**
Age >60 years	0.85 (0.60, 1.20)	0.3542			0.99 (0.55, 1.78)	0.9698		
Smoking	1.30 (0.91, 1.85)	0.1453			1.97 (0.87, 4.48)	0.1056		
BMI <18.5	0.94 (0.58, 1.53)	0.8120			1.21 (0.42, 3.47)	0.7238		
ECOG‐PS 2‐3	0.70 (0.40, 1.23)	0.2094			0.56 (0.17, 1.83)	0.3394		
Other histological type	1.50 (0.73, 3.08)	0.2695			1.09 (0.34, 3.55)	0.8830		
Stage IV	1.47 (0.88, 2.45)	0.1400			1.47 (0.73, 2.99)	0.2810		
Brain metastases	0.97 (0.62, 1.50)	0.8811			1.78 (0.82, 3.83)	0.1430		
Contralateral lung metastases	1.07 (0.76, 1.52)	0.6894			1.10 (0.58, 2.06)	0.7737		
Pleural metastases	1.19 (0.83, 1.72)	0.3409			1.07 (0.56, 2.05)	0.8322		
Pericardial metastases	1.12 (0.57, 2.21)	0.7459			1.53 (0.21, 11.31)	0.6770		
Liver metastases	1.53 (0.96, 2.46)	0.0755	1.10 (0.64, 1.89)	0.7389	2.10 (0.86, 5.11)	0.1038		
Adrenal metastases	1.52 (0.96, 2.42)	0.0752	1.08 (0.64, 1.82)	0.7641	1.78 (0.88, 3.59)	0.1074		
Bone metastases	1.26 (0.90, 1.78)	0.1837			1.83 (0.97, 3.43)	0.0612	1.78 (0.90, 3.53)	0.0997
Number of metastatic sites ≥2	1.79 (1.25, 2.55	**0.0013**	1.77 (1.19, 2.63)	**0.0045**	2.96 (1.57, 5.58)	**0.0008**	3.05 (1.55, 5.99)	**0.0012**
Taxanes ± platinum	1.0		1.0		1.0		1.0	
Pemetrexed ± platinum	0.62 (0.38, 1.02)	0.0617	0.63 (0.38, 1.06)	0.0825	0.44 (0.16, 1.21)	0.1117	0.43 (0.14, 1.25)	0.1213
Chemotherapy + antiangiogenesis/immunotherapies	0.50 (0.28, 0.91)	**0.0222**	0.49 (0.27, 0.91)	**0.0233**	0.34 (0.12, 0.98)	**0.0466**	0.24 (0.08, 0.75)	**0.0145**
KRAS WT	1.0							
KRAS mutation	0.94 (0.66, 1.35)	0.7461						
KRAS G12C	1.07 (0.55, 2.06)	0.8443						
KRAS non‐G12C	0.94 (0.54, 1.66)	0.8422						

*Note*: Independent variables with *p* < 0.10 in the univariate analyses were included in the Cox proportional hazards model. *p* values < 0.05 were highlighted in bold.

Abreviations: BMI, body mass index; CI, confidence interval; ECOG PS, Eastern Cooperative Oncology Group Performance Status; HR, hazard ratio; WT, wild‐type.

^a^ Univariate and multivariate analysis for PFS in all patients after first‐line treatment;

^b^ Univariate and multivariate analysis for PFS in KRAS‐mutated patients after first‐line treatment.

### 3.3 Effect of KRAS mutation on OS

The outcomes were illustrated in Table 4. Univariate analyses indicated that KRAS mutation was closely correlated with a poorer median OS than KRAS WT (HR = 2.36; 95% CI, 1.55–3.61; *p* < 0.0001), and the median OS time was 9.9 and 21.5 months for the KRAS mutant and KRAS WT groups, respectively (Figure 2B). Compared with the KRAS WT patients, the patients harboring KRAS rare mutations had the shortest OS time, followed by G12V and G12D (Figure 2D). The median OS in KRAS rare mutation, G12V and G12D groups were 8.3, 9.3 and 12.5 months, respectively. After adjustment for smoking status, liver metastases, adrenal metastases, and number of metastatic sites, KRAS mutations (HR = 2.39, 95% CI, 1.53–3.73, *p* = 0.0001) and KRAS non‐G12C (HR = 4.10, 95% CI, 2.32–7.25, *p* < 0,0001) remained significantly associated with increased risk of death. Regrettably, no significant difference in OS was observed among KRAS mutant patients with different first‐line treatments (Figure 2F).

**TABLE 4:** Univariate and multivariate analyses of potential predictive factors of death hazard.

	^a^Univariate analyses HR (95% CI)	*p*‐value	^a^Multivariate analyses HR (95% CI)	*p*‐value	^b^Univariate analyses HR (95% CI)	*p*‐value	^b^Multivariate analyses HR (95% CI)	*p*‐value
Male	1.40 (0.86, 2.30)	0.1761			2.41 (0.84, 6.95)	0.1034		
Age >60 years	1.23 (0.81, 1.86)	0.3417			0.88 (0.48, 1.62)	0.6838		
Smoking	1.62 (1.05, 2.50)	**0.0287**	1.38 (0.84, 2.27)	0.2084	1.55 (0.71, 3.40)	0.2716		
BMI <18.5	0.97 (0.54, 1.75)	0.9151			0.93 (0.32, 2.72)	0.8911		
ECOG‐PS 2‐3	1.03 (0.50, 2.13)	0.9442			0.95 (0.28, 3.25)	0.9293		
Other histological type	0.82 (0.33, 2.02)	0.6629			0.44 (0.11, 1.85)	0.2647		
Stage IV	1.00 (0.56, 1.80)	0.9971			1.84 (0.84, 4.01)	0.1267		
Brain metastases	0.93 (0.53, 1.65)	0.8035			1.53 (0.72, 3.21)	0.2660		
Contralateral lung metastases	1.26 (0.82, 1.94)	0.2967			1.13 (0.57, 2.27)	0.7243		
Pleural metastases	1.02 (0.66, 1.58)	0.9215			1.12 (0.59, 2.13)	0.7318		
Pericardial metastases	0.77 (0.28, 2.10)	0.6079			0.00 (0.00, Inf)	0.9976		
Liver metastases	1.70 (0.98, 2.94)	0.0587	1.22 (0.65, 2.29)	0.5260	1.83 (0.84, 3.99)	0.1308		
Adrenal metastases	1.76 (1.04, 3.00)	**0.0357**	1.14 (0.62, 2.11)	0.6658	2.30 (1.12, 4.72)	**0.0232**	5.43 (1.71, 17.23)	**0.0040**
Bone metastases	1.20 (0.79, 1.83)	0.3993			1.79 (0.95, 3.38)	0.0728	1.16 (0.35, 3.85)	0.8065
Number of metastatic sites ≥2	1.83 (1.20, 2.79)	**0.0047**	1.92 (1.17, 3.14)	**0.0096**	1.98 (1.08, 3.65)	**0.0282**	1.58 (0.43, 5.83)	0.4935
Taxanes ± platinum	1.0				1.0			
Pemetrexed ± platinum	1.05 (0.56, 1.97)	0.8701			0.80 (0.27, 2.40)	0.6971		
Chemotherapy + antiangiogenesis/immunotherapies	1.04 (0.47, 2.29)	0.9208			0.68 (0.20, 2.27)	0.5302		
KRAS WT	1.0		1.0					
KRAS mutation	2.36 (1.55, 3.61)	**<0.0001**	2.39 (1.53, 3.73)	**0.0001**				
KRAS G12C	1.76 (0.86, 3.60)	0.1225	1.78 (0.83, 3.78)	0.1361	1.0		1.0	
KRAS non‐G12C	3.45 (1.98, 6.00)	**<0.0001**	4.10 (2.32, 7.25)	**<0.0001**	2.15 (0.93, 5.00)	0.0738	2.27 (0.82, 6.33)	0.1160

*Note*: Independent variables with *p* < 0.10 in the univariate analyses were included in the Cox proportional hazards model. *p* values < 0.05 were highlighted in bold.

Abbreviations: BMI, body mass index; CI, confidence interval; ECOG PS, Eastern Cooperative Oncology Group Performance Status; HR, hazard ratio; WT, wild‐type.

^a^ Univariate and multivariate analysis for death hazard in all patients after first‐line treatment.

^b^ Univariate and multivariate analysis for death hazard in KRAS‐mutated patients after first‐line treatment.

### 3.4 Stratified analyses by potential confounders

**FIGURE 3:**
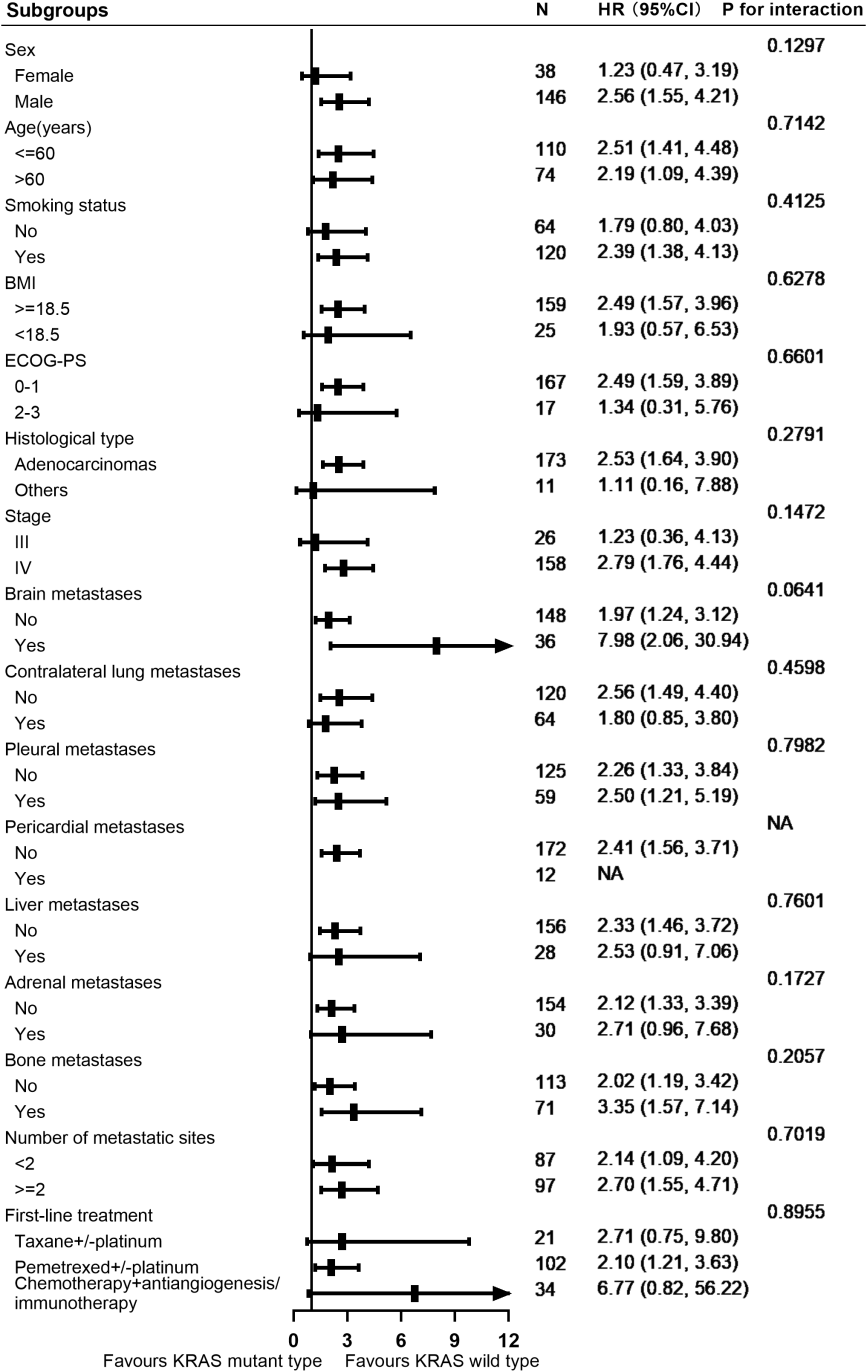
Forest plot of overall survival subgroup analyses.

To further clarify that the correlation between KRAS status and risk of death in non‐squamous NSCLC patients were robust to important covariates, we performed stratified analyses for subgroups, including sex, age, smoking status, ECOG PS, BMI, histological type, metastatic organs, number of metastatic sites, clinical stage, and first‐line treatment. Figure 3 further confirmed a highly consistent pattern: among patients with non‐squamous NSCLC, regardless of any subgroup mentioned above, KRAS mutation resulted in a significant increase in risk of mortality. Correspondingly, in interaction tests for potential effect modification, no significant interaction effect was observed.

## 4 DISUSSION

With the introduction of detecting oncogenic driver mutations, we have significantly improved the overall survival of non‐small cell lung cancer patients with targetable mutations. Precision medicine, which is currently advocated for cancer treatment, requires molecular analysis of tumor tissue before treatment to select the best therapeutic regimen. However, effective targeted therapies for KRAS mutations have not been successfully developed to date. Platinum‐based chemotherapy remains the priority selection for patients with KRAS mutations. Since KRAS mutations were first considered as adverse prognostic factors of lung cancer in the 1990s,^21^ the prognostic and predictive value of KRAS mutations in lung carcinoma has been always a topic of controversial discussion, though a lot of clinical studies investigating KRAS mutations have been published in recent decades. Considering the differences of these studies in population, race, region, sample size, histological type, tumor stage, composition of genetic mutation type, and first‐line treatment, a consistent conclusion was difficult to draw.

We reviewed the literature about the impact of KRAS mutations on survival in advanced NSCLC patients receiving platinum‐based chemotherapy and founds that many articles reported inconsistent results. Loriot et al reviewed all relevant literatures on the relationship between clinical outcomes and advanced NSCLC patients with KRAS mutations using conventional chemotherapy and concluded that KRAS mutations had no values in predicting response to conventional chemotherapy.^22^ A retrospective investigation conducted by Mellema et al. suggested that NSCLC patients with KRAS mutations had similar clinical response to chemotherapy and median OS compared to those without KRAS mutations.^23^ Results from TAILOR trial also indicated that KRAS mutations had no negative effects on PFS and OS in NSCLC.^24^ In contrast, a study by Metro et al. involved patients with advanced non‐squamous EGFR wild‐type NSCLC, and demonstrated that KRAS mutation was significantly associated with lower response rates to chemotherapy and inferior PFS.^25^ Furthermore, Hames et al. reported that KRAS mutations conferred advanced NSCLC patients treated with platinum‐based chemotherapy to a worse prognosis compared to KRAS wild‐type.^26^

In this study, KRAS mutations were neither significantly associated with response rates of first‐line treatments nor risk for disease progression in advanced NSCLC, but the independent effect of KRAS mutations on overall survival was observed. Notably, the proportion of missing treatment information was higher in KRAS MT patients than that in KRAS WT patients. Possible selection bias can affect overall survival in patients with KRAS mutations. Therefore, we included first‐line treatment in the multivariate analysis for the risk of death to minimize the effect on overall survival. The results indicated that KRAS mutations (HR = 2.32, 95% CI, 1.45–3.70, *p* = 0.0004) and KRAS non‐G12C (HR = 2.98, 95% CI, 1.55–5.74, *p* = 0.0011) remained significantly associated with an increased risk of death after adjustment for treatment, smoking status, liver metastases, adrenal metastases, and number of metastatic sites. Moreover, the positive relationship between KRAS mutations and mortality hazard was stable in all stratified populations, and no modification effect of other confounding factors and special population were found, indicating the solidity and low heterogeneity of our conclusions. The current study verified that the resistance mechanisms and patterns for first‐line therapy in KRAS MT and WT patients might be similar, while the correlation between KRAS mutation and poor overall survival of non‐squamous NSCLC was based on the hypothesis that KRAS as oncogenic driver mutation constantly to activate multiple downstream signaling pathways, such as RAF/MEK/ERK, PI3K/AKT/mTOR, NF/κB, etc. Promoting tumor survival, anti‐apoptosis, proliferation, angiogenesis, invasion, and metastasis, leading to rapidly disease progression and short survival.^17^, ^27^

Among the KRAS mutation types, non‐G12C mutations were independent risk factors for overall survival. Although Ihle et al. confirmed that G12V mutation significantly affected PFS, they did not further explore the relationship between G12V and OS. This study first revealed the independent association between KRAS non‐G12C mutations and death hazard in Chinese patients with advanced non‐squamous NSCLC. Previous literatures have shown that KRAS G12D and G12V promote the evolution and formation of lung adenocarcinoma.^28^, ^29^ Specific changes of different positions and amino acids of KRAS protein had different effects on the downstream signaling pathway, and then lead to different biological characteristics and transformation ability.^30^ Therefore, detection of specific KRAS mutant subtypes should be completed in clinical practice, which may help to determine the optimal treatment option for individuals.

In addition, multivariate analysis demonstrated that chemotherapy regimens including immunotherapy or antiangiogenic therapy were significantly related with the decline in risk of disease progression regardless of KRAS status. In terms of PFS, patients with KRAS mutations were likely to benefit more from chemotherapy in combination with immunotherapy or antiangiogenic therapy than those of KRAS WT. Reviewing the previous literatures, these conclusions may be the first to be confirmed in the Chinese non‐squamous NSCLC population. Vascular endothelial growth factor (VEGF) is a pro‐angiogenic factor that may play an essential role in tumor progression and metastasis. KRAS‐mutated tumors can mediate the production of VEGF and interleukin‐8 through MAPK and NF‐κB pathway in KRAS mutations, and then promote the formation of tumor blood vessels.^31^, ^32^ Inhibitive therapies targeting VEGF inhibit the new tumor vascular system, shrink the existing tumor microvascular, and normalize the remaining tumor vascular system. The addition of antiangiogenic therapy may affect the upregulated tumor angiogenesis signaling pathway and enhance the antitumor efficacy. On the other hand, recent evidence demonstrated that KRAS mutant lung cancer exhibited immune‐suppressive and inflammatory tumor microenvironments to promote tumor progression through some molecular biological mechanisms. Coelho et al. reported an mRNA binding protein called tristetraprolin (TTP) that could bind to mRNA translating PD‐L1 and accelerate PD‐L1 mRNA degradation to increase anti‐tumor immunity. However, KRAS‐mutated lung cancers inhibited TTP through activation of MEK downstream signaling and augmented PD‐L1 expression, thus presenting immunoresistant phenotype.^33^ KRAS mutation was also associated with tumor lymphocytes infiltration and increased tumor mutation burden in NSCLC except for high PD‐L1 expression in tumor cells, resulting in better efficacy of PD‐1/PD‐L1 inhibitors. Moreover, three meta‐analyses further confirmed that patients with KRAS MT got more clinical benefit in anti‐PD‐1 /PD‐L1 immunotherapy than those with KRAS WT.^34^ These findings support the results we have observed so far. It is a pity that we did not observe any difference in overall survival between first‐line treatment regimens in KRAS‐mutated patients, but there was a tendency to improve long‐term survival in patients with KRAS mutation using immunotherapy or antiangiotherapy in combination with chemotherapy, and we expect surprising findings from larger sample sizes.

Several limitations should be acknowledged in the current study. First, due to the nature of retrospective studies, selection bias is inevitable. Second, since the study was designed in a single institution, whether our findings can be extended to other ethnic populations may require additional studies to validate. Third, the small sample size is also a shortcoming of this study. In view of the low mutation prevalence in the Chinese population, we believe that the sample size is within an acceptable range. Fourth, the missing amino acid substitution information in 41 patients with KRAS mutation may bias the distribution of KRAS subtypes. Considering that the amino acid substitution distribution of the other 35 cases was similar to that reported by previous publications,^5^, ^35^ the overall risk of bias of the distribution was modest. Finally, Considering the low incidence of KRAS mutations in Chinese population, we only investigated whether KRAS mutations were a negative predictor of PFS and OS in Chinese patients with advanced non‐squamous NSCLC after first‐line treatment. Further studies are needed to compare the responses of different KRAS subtypes to first‐line treatment in advanced non‐squamous NSCLC.

## 5 CONCLUSION

In conclusion, KRAS mutation and its subtypes were not independent risk factors for predicting the risk of disease progression, but KRAS mutation and KRAS non‐G12C were significantly associated with an increased risk of death. In addition, first‐line chemotherapy combined with antivascular therapy or immunotherapy was significantly associated with a reduced risk of disease progression regardless of KRAS mutation status. Patients with KRAS MT may benefit even more from chemotherapy combined with antivascular therapy or immunotherapy than those with KRAS WT.

## AUTHOR CONTRIBUTIONS

**Feiwen Liu:** Conceptualization (equal); data curation (equal); formal analysis (equal); methodology (equal); software (equal); visualization (equal); writing – original draft (equal). **Fang Wang:** Formal analysis (equal); methodology (equal); software (equal); writing – original draft (equal). **Jianbo He:** Data curation (equal); formal analysis (equal); resources (equal). **Shaozhang Zhou:** Funding acquisition (equal); investigation (equal); methodology (equal); project administration (equal); resources (equal); supervision (equal). **Min Luo:** Funding acquisition (equal); investigation (equal); methodology (equal); project administration (equal); resources (equal); supervision (equal).

## FUNDING INFORMATION

The research funding was provided by a “139 Talent Planning” granted by Guangxi Health Commission (grant number: 201903030) and the Natural Science Foundation of Guangxi Zhuang Autonomous Zone (grant number: 2015GXNSFAA139162), China.

## CONFLICT OF INTEREST STATEMENT

The authors declare no conflicts of interest regarding this study.

## Supporting information


Figure S1

## Data Availability

I confirm that my article contains a Data Availability Statement even if no data is available (list of sample statements) unless my article type does not require one. I confirm that I have included a citation for available data in my references section, unless my article type is exempt.
