# Supplementary figures and images for: Correlation between KRAS mutation subtypes and prognosis in Chinese advanced non‐squamous non‐small cell lung cancer patients
Source: Cancer Med. 2023 May 4;12(12):13123–34. doi: 10.1002/cam4.5995 (PMC10315828; doi:10.1002/cam4.5995)

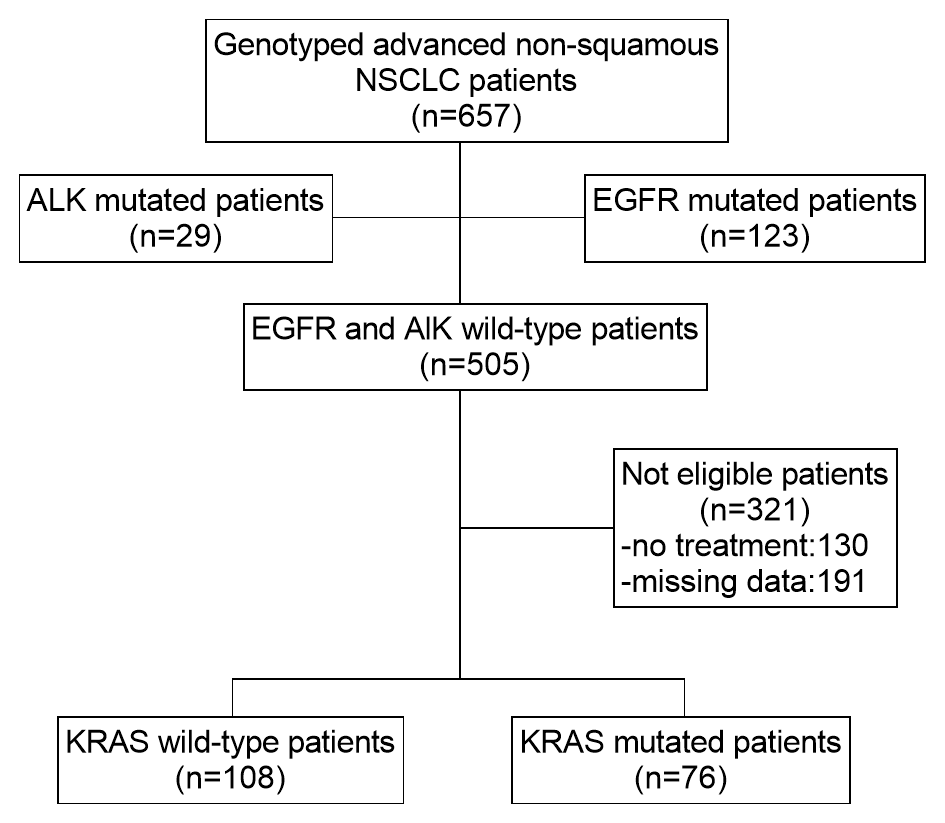

Supplement: Supplementary file 1 — Figure S1 [file CAM4-12-13123-s001.tif]
